# Alterations of oral and gut viromes in hypertension and/or periodontitis

**DOI:** 10.1128/msystems.01169-23

**Published:** 2023-12-18

**Authors:** Hui-Lin Ye, Meng-Fan Zhi, Bo-Yan Chen, Wen-Zhen Lin, Yu-Lin Li, Shi-Jia Huang, Lu-Jun Zhou, Shuo Xu, Jun Zhang, Wu-Chang Zhang, Qiang Feng, Sheng-Zhong Duan

**Affiliations:** 1 Laboratory of Oral Microbiota and Systemic Diseases, Shanghai Ninth People’s Hospital, College of Stomatology, Shanghai Jiao Tong University School of Medicine, Shanghai, China; 2 National Center for Stomatology, Shanghai Key Laboratory of Stomatology, Shanghai, China; 3 National Clinical Research Center for Oral Diseases, Shanghai Key Laboratory of Stomatology, Shanghai, China; 4 Department of Human Microbiome, School and Hospital of Stomatology, Cheeloo College of Medicine, Shandong University & Shandong Key Laboratory of Oral Tissue Regeneration & Shandong Engineering Laboratory for Dental Materials and Oral Tissue Regeneration & Shandong Provincial Clinical Research Center for Oral Diseases, Jinan, China; NYU Langone Health, New York, New York, USA

**Keywords:** oral virome, gut virome, hypertension, periodontitis, oral-gut viral correlations, virus-bacterium interactions

## Abstract

The microbiota plays an important role in both hypertension (HTN) and periodontitis (PD), and PD exacerbates the development of HTN by oral and gut microbiota. Previous studies have focused on exploring the importance of the bacteriome in HTN and PD but overlooked the impact of the virome, which is also a member of the microbiota. We collected 180 samples of subgingival plaques, saliva, and feces from a cohort of healthy subjects (nHTNnPD), subjects with HTN (HTNnPD) or PD (PDnHTN), and subjects with both HTN and PD (HTNPD). We performed metagenomic sequencing to assess the roles of the oral and gut viromes in HTN and PD. The HTNnPD, PDnHTN, and HTNPD groups all showed significantly distinct beta diversity from the nHTNnPD group in saliva. We analyzed alterations in oral and gut viral composition in HTN and/or PD and identified significantly changed viruses in each group. Many viruses across three sites were significantly associated with blood pressure and other clinical parameters. Combined with these clinical associations, we found that *Gillianvirus* in subgingival plaques was negatively associated with HTN and that *Torbevirus* in saliva was positively associated with HTN. We found that *Pepyhexavirus* from subgingival plaques was indicated to be transferred to the gut. We finally evaluated viral-bacterial transkingdom interactions and found that viruses and bacteria may cooperate to affect HTN and PD. Correspondingly, HTN and PD may synergize to improve communications between viruses and bacteria.

**IMPORTANCE:**

Periodontitis (PD) and hypertension (HTN) are both highly prevalent worldwide and cause serious adverse outcomes. Increasing studies have shown that PD exacerbates HTN by oral and gut microbiota. Previous studies have focused on exploring the importance of the bacteriome in HTN and PD but overlooked the impact of the virome, even though viruses are common inhabitants in humans. Alterations in oral and gut viral diversity and composition contribute to diseases. The present study, for the first time, profiled the oral and gut viromes in HTN and/or PD. We identified key indicator viruses and their clinical implications in HTN and/or PD. We also investigated interactions between viruses and bacteria. This work improved the overall understanding of the viromes in HTN and PD, providing vital insights into the role of the virome in the development of HTN and PD.

## INTRODUCTION

Periodontitis (PD) is one of the most common oral diseases, impacting 796 million people and causing large direct and indirect costs globally (1, 2). Hypertension (HTN) is also highly prevalent across the worldwide and has a significant influence on cardiovascular disease complications and even death. Increasing evidence suggests that PD is closely associated with an increased prevalence of hypertension and linked to higher systolic blood pressure (SBP) and/or diastolic blood pressure (DBP) levels (3, 4). Moreover, periodontal treatment contributes to improving endothelial function and reducing inflammatory markers, SBP and DBP (5–7).

Immune and inflammatory mechanisms play an important role in the development of HTN (8). PD causes dysbiosis of the oral microbiome and immune defenses of the host, leading to the systemic spread of oral microbiota and inflammation (9). Previous studies have indicated that PD may aggravate HTN via systemic inflammation. Recent studies have revealed that dysfunctional oral and gut microbiotas play a prominent role in HTN (10, 11). The oral and gut microbiotas of hypertensive patients are quite different from those of healthy subjects (12, 13). The abundance of nitrate-reducing bacteria is lower in patients with PD (14). *Porphyromonas gingivalis*, a common PD pathogen, has been reported to exacerbate angiotensin II-induced hypertension and vascular dysfunction (15, 16). Many studies have suggested that the gut microbiota and its metabolites play a role in the regulation of blood pressure (BP) (17). PD substantially influences the gut microbiome by hematogenous routes or swallowing pathogens and related metabolites, which may trigger metabolic endotoxemia and systemic inflammation, further contributing to HTN (18, 19).

The associations among bacteria, HTN, and PD have been extensively investigated, but few studies have focused on the contribution of viruses to HTN and PD. Viruses and bacteria are both important components of the microbiota, and viruses are the most diverse and abundant parasites in the human body (20). The virome mainly consists of animal-infecting viruses and bacteriophages. Bacteriophages have been reported to account for 20%–80% of total bacterial death by infecting bacteria, indicating that the virome may play a profound role in limiting the bacterial population (21). Previous studies have found that the composition of gut viruses is significantly changed in HTN and that gut viruses have a superior resolution and discrimination ability compared with bacteria in distinguishing healthy samples and hypertensive samples (22). Some studies have reported that the prevalence of Epstein‒Barr virus and cytomegalovirus is significantly higher in the subgingival plaques of PD patients, and the prevalence of viruses is the highest in PD patients with coronary artery disease (23, 24). Overall, there is a lack of research on the role of the virome in HTN and PD.

Here, we collected 180 oral (subgingival plaques and saliva) and fecal samples and performed metagenomic sequencing, aiming to profile the oral and gut viromes in HTN and PD as well as to link the virome with HTN and PD. We further investigated oral-gut viral communication and explored the correlations between viruses and bacteria, expecting to provide important information for virome studies in HTN and PD.

## RESULTS

### Altered diversity of oral and gut viromes in patients with HTN and/or PD

**Fig 1:**
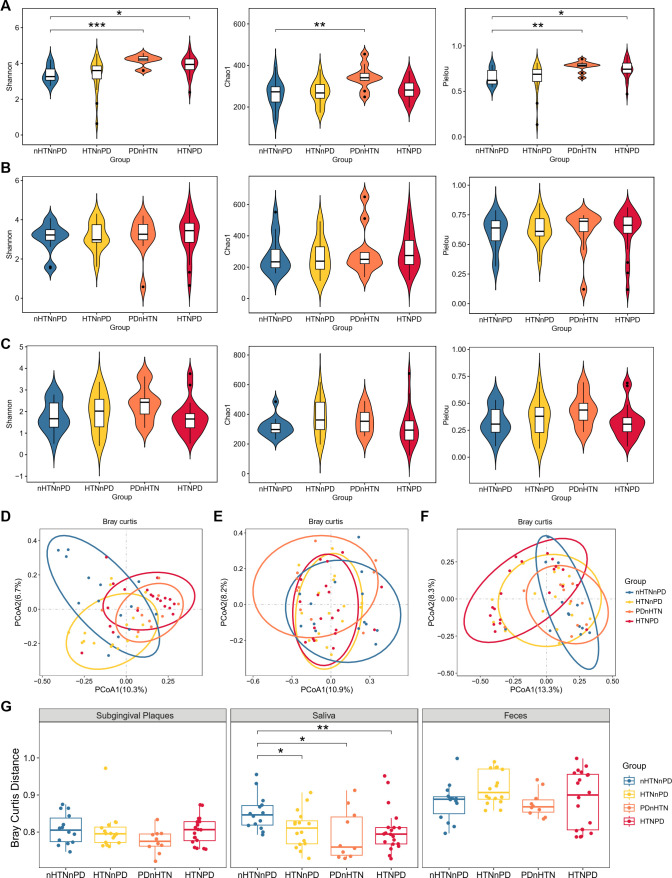
Altered diversity of oral and gut virome in patients with HTN and/or PD. (**A–C**) Shannon index, Chao1 richness, and Pielou evenness of virome in subgingival plaques (**A**), saliva (**B**), and feces (**C**) of participants with or without HTN/PD. (**D–F**) PCoA of virome in subgingival plaques (**D**), saliva (**E**), and feces (**F**). (**G**) Bray–Curtis distance of oral (subgingival plaques and saliva) and gut virome in participants with or without HTN/PD. *n* = 14, 16, 10, and 20 for the nHTNnPD, HTNnPD, PDnHTN, and HTNPD groups, respectively. Wilcoxon rank-sum test was used for statistical analysis in A–C and G. *P < 0.05, **P < 0.01, ***P < 0.001.

To investigate the impact of HTN and PD on oral and gut viromes, we collected subgingival plaques, saliva, and fecal samples from 60 participants, including 14 healthy individuals (nHTNnPD), 16 participants with HTN but without PD (HTNnPD), 10 people with PD but without HTN (PDnHTN), and 20 participants with both HTN and PD (HTNPD). The detailed demographics and clinical parameters of these participants are presented in Table S1 in the Supplemental material. We performed metagenomic sequencing for these samples. According to the results of alpha diversity, the HTNPD group showed an increased Shannon diversity and Pielou evenness index of the virome compared with the nHTNnPD group in the oral cavity (Fig. 1A and B), especially in the subgingival plaques, with a significant increase (Fig. 1A). Similarly, the PDnHTN group showed significantly higher Shannon diversity, Pielou evenness, and Chao1 richness compared with nHTNnPD controls in the subgingival plaques (Fig. 1A). In the gut, the alpha diversity of the other three groups did not show a significant difference from that of the nHTNnPD group (Fig. 1C). Additionally, we analyzed beta diversity using principal coordinates analysis (PCoA) for the oral and gut viromes (Fig. 1D through F). Based on Bray‒Curtis distance, the saliva virome composition of the other three groups was significantly separated from that of the nHTNnPD group (Fig. 1G). The beta diversity of the other three groups in subgingival plaques and gut viromes was not significantly different from that of the nHTNnPD group (Fig. 1G).

### Alterations of oral and gut viral compositions in patients with HTN and/or PD

**Fig 2:**
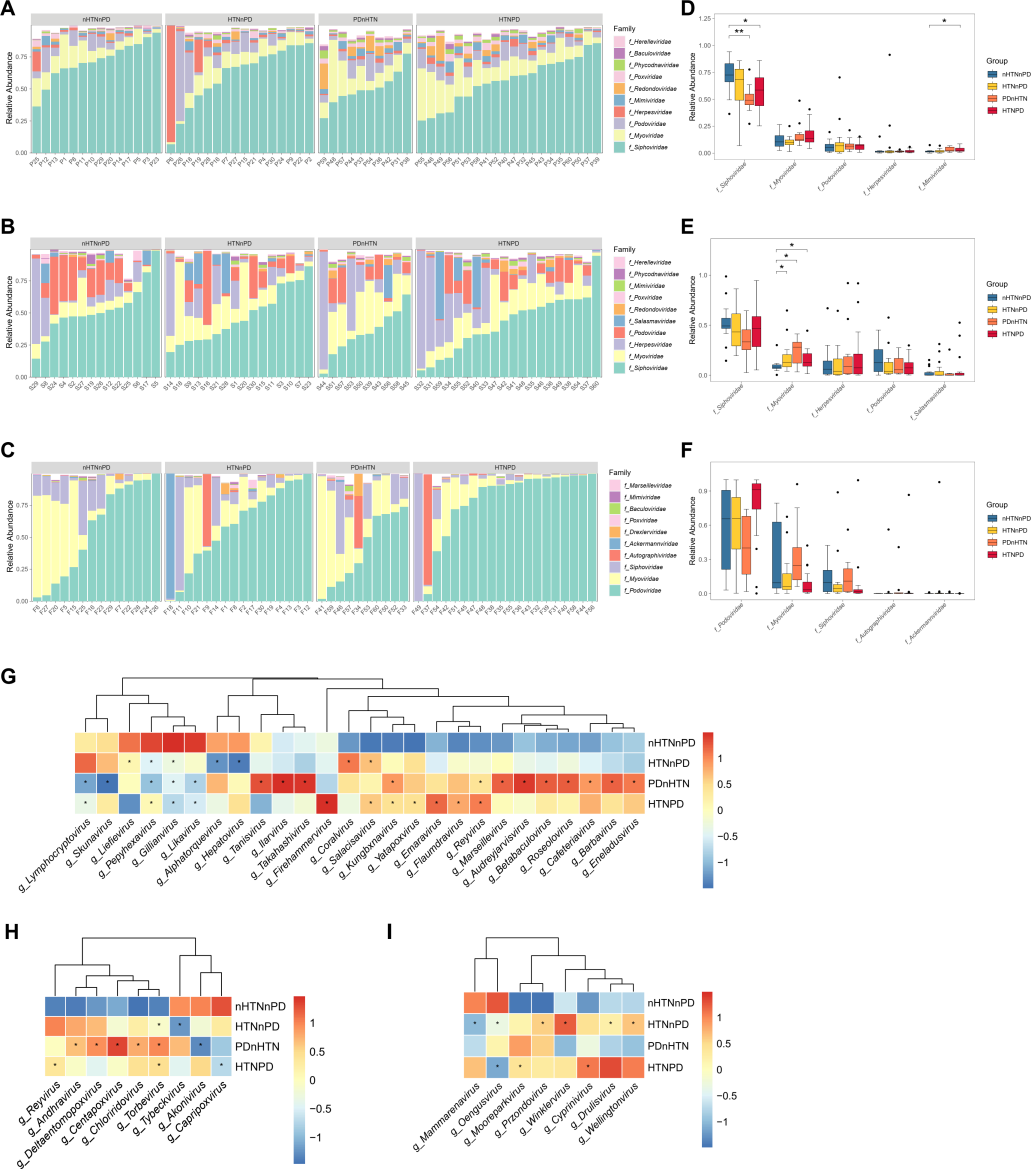
Alterations of oral and gut viral composition in patients with HTN and/or PD. (**A–C**) Stacked bar plots showing relative abundances of viruses at the family level in subgingival plaques (**A**), saliva (**B**), and feces (**C**) of participants with or without HTN/PD. (**D–F**) Analyses of relative abundances of the top 5 viral families in subgingival plaques (**D**), saliva (**E**), and feces (**F**). (**G–I**) Clustering heatmaps of relative abundances of viral genera in subgingival plaques (**G**), saliva (**H**), and feces (**I**) of participants with or without HTN/PD. *n* = 14, 16, 10, and 20 for the nHTNnPD, HTNnPD, PDnHTN, and HTNPD groups, respectively. Wilcoxon rank-sum test was used for statistical analysis in D–F. *P < 0.05, **P < 0.01, ***P < 0.001. Limma voom, MaAsLin2, and Wilcoxon test were used in G–I. *P < 0.05.

We then analyzed the compositions of the oral and gut viromes at the family level and found significant differences among the groups (Fig. 2A through C). Four of the top 5 viral families in subgingival plaques and saliva were identical, namely, *Siphoviridae*, *Myoviridae*, *Podoviridae*, and *Herpesviridae*. In addition, *Mimiviridae* and *Salasmaviridae* were also predominant families in subgingival plaques and saliva, respectively. *Podoviridae*, *Myoviridae*, *Siphoviridae*, *Autographiviridae*, and *Ackermannviridae* were prevalent in the fecal virome. Compared with healthy controls, the relative abundances of *Siphoviridae* and *Mimiviridae* were significantly changed in the subgingival plaques of HTNPD groups (Fig. 2D). *Siphoviridae* was significantly decreased in the subgingival plaques of PDnHTN group (Fig. 2D). In saliva, *Myoviridae* was significantly increased after subjects suffered from HTN and/or PD (Fig. 2E). In feces, no significant changes were found among the three groups (Fig. 2F). Other significant changes were also present in the top 6–10 viral families, mainly in subgingival plaques and saliva (Fig. S1).

We further visualized the compositions of the viromes at the genus level, and many differentially abundant viruses were identified among these groups. Among these three sites, the virome of the subgingival plaques was the most diversified in the four groups. The virome of the PDnHTN group was the most different from that of the nHTNnPD group with 12 significantly increased genera and 5 significantly decreased genera (Fig. 2G). Compared with the HTNnPD group, the HTNPD group showed more significantly changed genera. *Gillianvirus*, *Pepyhexavirus*, and *Salacisavirus* were significantly changed in both the HTNnPD and HTNPD groups (Fig. 2G). In the saliva virome, *Torbevirus* was augmented in the HTNnPD group, while *Tybeckvirus* was decreased in the HTNnPD group (Fig. 2H). In the HTNPD group, the relative abundance of *Reyvirus* was significantly increased in both subgingival plaques and saliva (Fig. 2G and H), suggesting its important role in the oral virome of the HTNPD group. In feces, the most changes in the virome occurred in the HTNnPD group, with four genera increasing and two genera decreasing (Fig. 2I). There were three genera in the HTNPD group but no genus in the PDnHTN group that showed significant differences compared with the nHTNnPD group.

### Distinct core oral and gut viromes in HTN and PD

**Fig 3:**
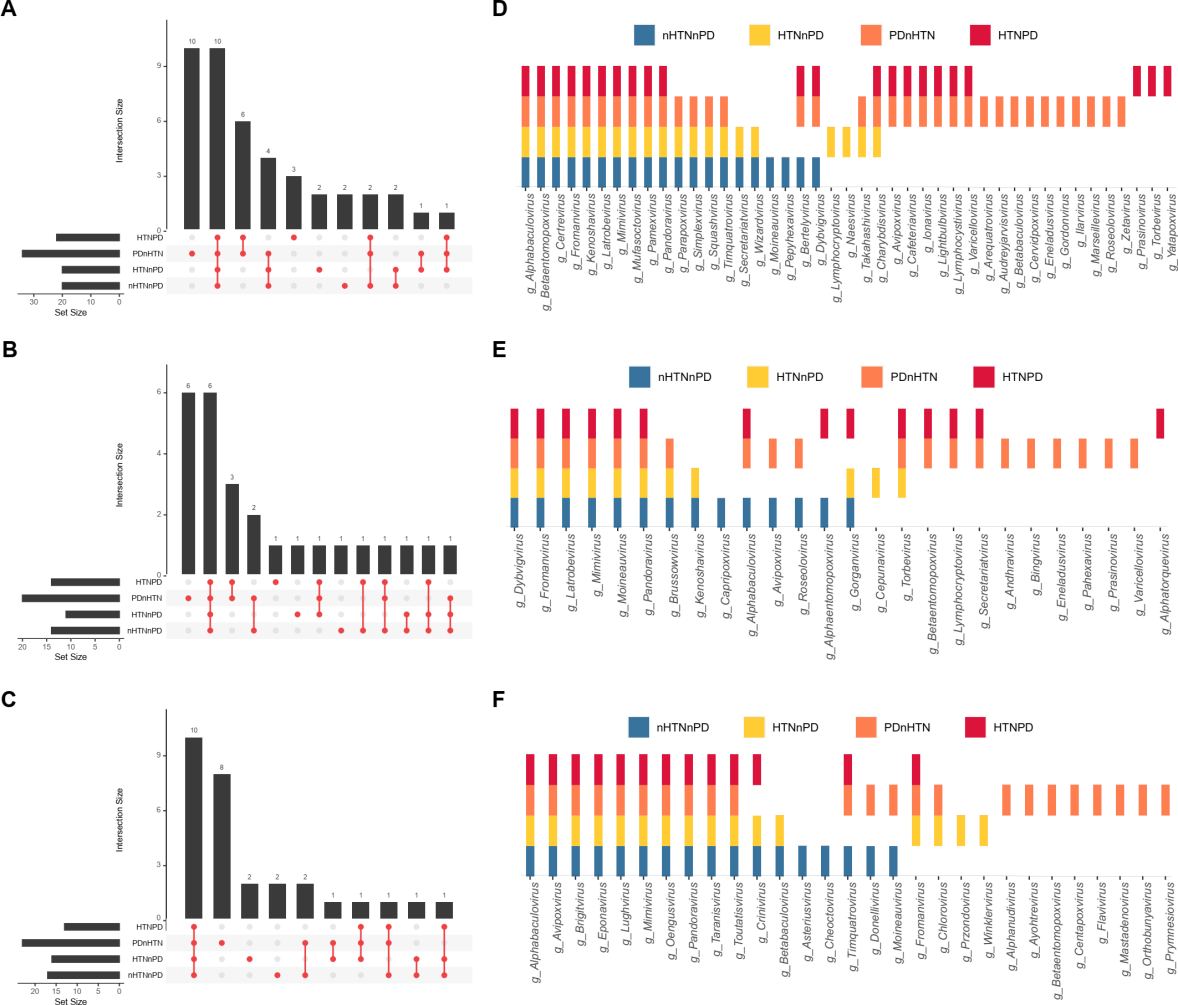
Distinct core oral and gut virome in HTN and PD. (**A–C**) Core viral genera identified in subgingival plaques (**A**), saliva (**B**), and feces (**C**) of participants with or without HTN/PD. (**D–F**) Comparisons of the core viral genera in subgingival plaques (**D**), saliva (**E**), and feces (**F**). *n* = 14, 16, 10, and 20 for the nHTNnPD, HTNnPD, PDnHTN, and HTNPD groups, respectively.

We identified the core oral and gut viromes and found that most of the core viromes were shared by the four groups at the same site, and some of the core viromes only existed in a specific group (Fig. 3A through C). The PDnHTN group had the most core viromes, which, to some extent, was consistent with its high alpha diversity (Fig. 1A through C). The detailed distribution of the core viromes is presented by bar plots. In subgingival plaques, the mutual 10 genera comprised the core viromes of these four groups (Fig. 3D). *Lymphocryptovirus* and *Naesvirus* were identified as the core viromes only in the HTNnPD group. Most of the core viromes in the HTNPD group were identical to those of the PDnHTN group, indicating an influence of PD on the subgingival plaques core viromes. In saliva, 8 out of the 14 core viromes in the nHTNnPD group, 6 out of the 11 core viromes in the HTNnPD group, 14 out of the 20 core viromes in the PDnHTN group, and 8 out of the 14 core viromes in the HTNPD group were identical to the core viromes of the same group in subgingival plaques (Fig. 3E). Unlike the similarity between subgingival plaques and saliva, the core viromes of feces differed from the oral core viromes (Fig. 3F). A total of 17 genera constituted the core viromes of the nHTNnPD group, and 16 genera formed the core viromes of the HTNnPD group with 12 identical core viromes of the nHTNnPD group. In total, 23 genera comprised the core viromes of the PDnHTN group, and 13 genera formed the core viromes of the HTNPD group with only one genus different from that of the PDnHTN group.

### Associations of oral and gut viromes with BP and other clinical parameters

**Fig 4:**
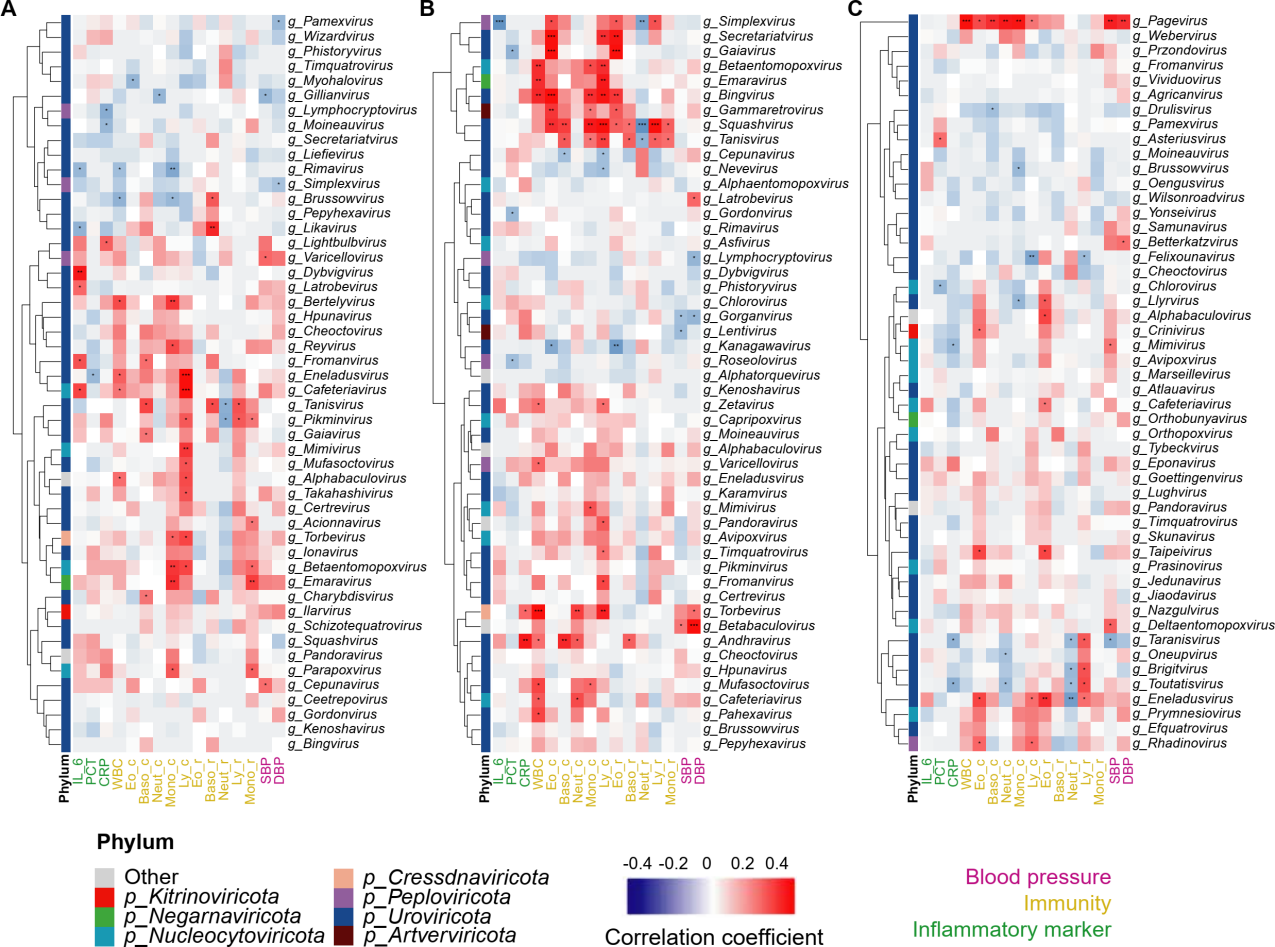
Associations between oral/gut virome and BP and other clinical parameters. (**A–C**) Heatmaps of Spearman’s correlation coefficients between clinical parameters (BP, immunity, and inflammatory markers) and relative abundances of the top 50 viral genera of subgingival plaques (**A**), saliva (**B**), and feces (**C**). IL-6, interleukin-6; PCT, procalcitonin; CRP, C-reactive protein; WBC, white blood cells; Eo_c, eosinophil count; Baso_c, basophil count; Neut_c, neutrophil count; Mono_c, monocyte count; Ly_c, lymphocyte count; Eo_r, eosinophil ratio; Baso_ r, basophil ratio; Neut_ r, neutrophil ratio; Mono_r, monocyte ratio; Ly_ r, lymphocyte ratio; SBP, systolic blood pressure; DBP, diastolic blood pressure. *n* = 60. *P < 0.05, **P < 0.01, ***P < 0.001.

To investigate the relationship between the oral/gut viromes and the clinical features of HTN as well as to elucidate their possible value for clinical diagnosis and treatment, we analyzed associations of BP and other clinical parameters with viruses in the top 50 genera in subgingival plaques, saliva, and feces. In subgingival plaques (Fig. 4A), SBP was positively correlated with *Varicellovirus* and *Cepunavirus* but negatively correlated with *Gillianvirus*, whereas DBP was negatively correlated with *Pamexvirus* and *Simplexvirus*. In saliva (Fig. 4B), *Betabaculovirus* was positively correlated with both SBP and DBP, while *Gorganvirus* was negatively correlated with both SBP and DBP. In saliva, *Lentivirus* was negatively correlated with SBP. In addition, *Latrobevirus* and *Torbevirus* were positively correlated with DBP in saliva, but *Lymphocryptovirus* was negatively correlated with DBP in saliva. In feces (Fig. 4C), *Pagevirus* was positively correlated with both SBP and DBP. Moreover, *Mimivirus* and *Deltaentomopoxvirus* were positively correlated with SBP in feces, and *Betterkatzvirus* was positively correlated with DBP in feces. Furthermore, *Taranisvirus* was negatively correlated with SBP in feces.

Because of the important role of inflammation in HTN, we also analyzed the correlations between the oral/gut viromes and HTN-related inflammatory indices. Interleukin-6 (IL-6) was significantly correlated with six subgingival genera and one salivary genus (Fig. 4A and B). Procalcitonin (PCT) was significantly correlated with one genus in subgingival plaques, three genera in saliva, and two genera in feces (Fig. 4A through C). C-reactive protein (CRP) was significantly correlated with three subgingival genera, two salivary genera, and three fecal genera (Fig. 4A through C). BP-related *Torbevirus* and *Taranisvirus* were also discovered to be significantly correlated with CRP.

Furthermore, we analyzed the associations of the oral and gut viromes with immune markers. In addition to the association with SBP and DBP, most subgingival and salivary viromes were significantly correlated with lymphocyte count and monocyte count, while most fecal viromes were correlated with lymphocyte ratio and eosinophil count (Fig. 4A through C). Moreover, *Mimivirus* and *Cafeteriavirus* in all three sites were significantly correlated with at least one BP-related clinical parameter (Fig. 4A through C), indicating their importance in the regulation of BP.

We also analyzed correlations of the oral and gut viromes with other clinical indices, including electrolytes, red blood cells, platelets, immunoglobulins, and hepatic and renal function (Fig. S2). Overall, more oral viromes were significantly correlated with BP-related clinical parameters than fecal viromes. Combined with the results of Fig. 2G through I, among those significantly changed viromes in HTNnPD and HTNPD, we concluded that *Gillianvirus* in subgingival plaques was negatively associated with HTN and that *Torbevirus* in saliva was positively associated with HTN. Because PD results in higher systemic levels of IL-6 and CRP (25) and combined with significantly changed genera in the PDnHTN group (Fig. 2G and H), we found that decreased *Likavirus* in subgingival plaques was negatively associated with IL-6 and that increased *Cafeteriavirus* was positively associated with IL-6. Increased *Torbevirus* and *Andhravirus* in saliva were positively associated with CRP. These results suggested an association of PD with *Likavirus*, *Cafeteriavirus*, *Torbevirus*, and *Andhravirus* (Fig. 4A and B).

### Communications between oral and gut viromes in participants with or without HTN and/or PD

**Fig 5:**
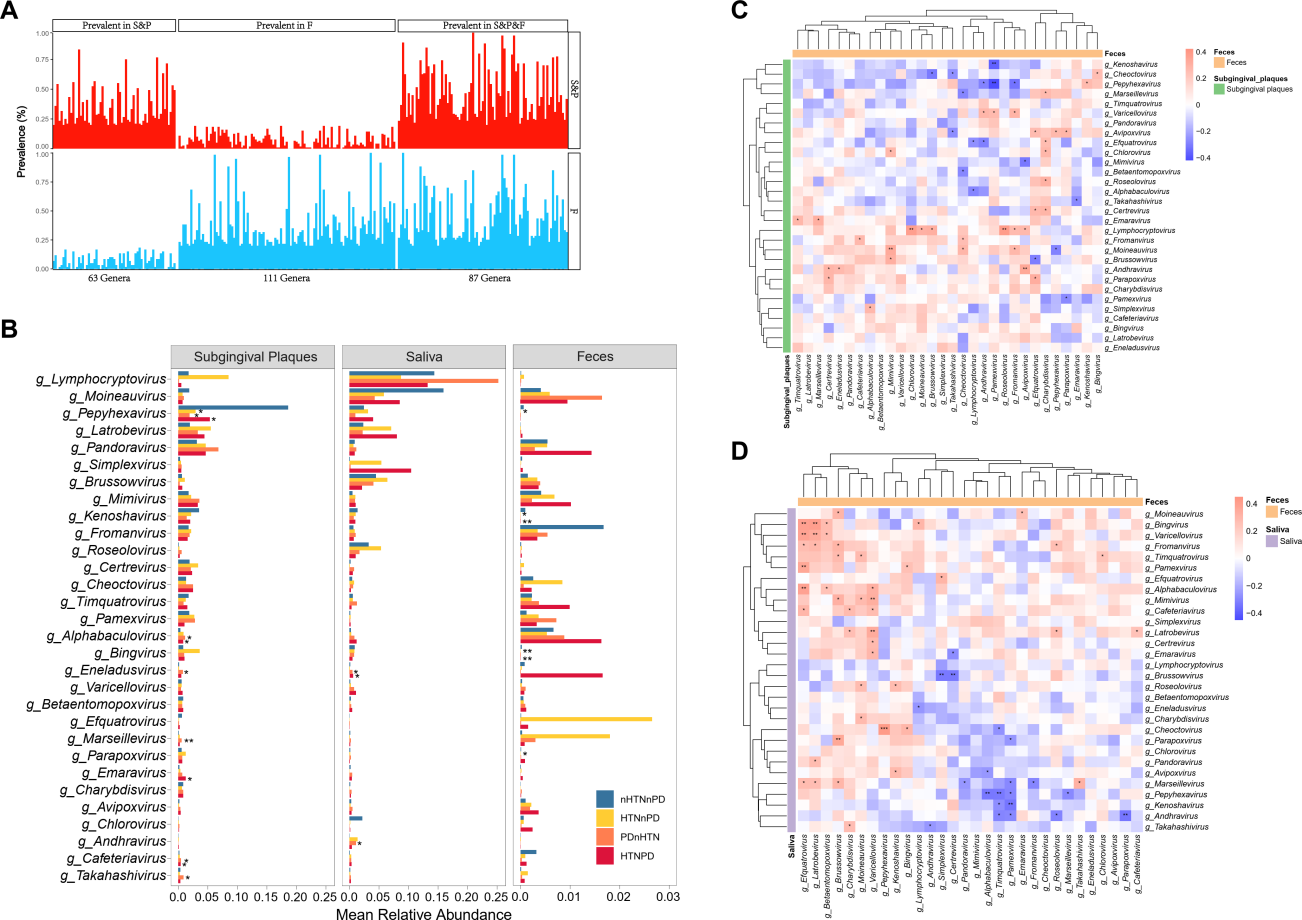
Communications between oral and gut virome in participants with or without HTN and/or PD. (**A**) Overview of viral genera prevalent in oral samples (saliva and subgingival plaques, abbreviated as S&P), gut samples (feces, abbreviated as F), or both oral and gut samples (saliva, subgingival plaques and gut, abbreviated as S&P&F). (**B**) Relative abundances of the top 30 genera prevalent in both oral and gut samples. (**C and D**) Clustering heatmaps of Spearman’s correlation coefficients between relative abundances of the top 30 shared genera in the gut and those in subgingival plaques (**C**) and saliva (**D**). *n* = 14, 16, 10, and 20 in B; *n* = 60 in A, C, and D. *P < 0.05, **P < 0.01, ***P < 0.001.

As a part of the digestive tract, the oral cavity and the gut are anatomically continuous, and interactions between the oral and gut microbiota occur frequently (26). Recent studies have suggested that the oral-gut microbial transmission is involved in many diseases (19, 27). Hence, we next assessed communications between the oral and gut viromes in HTN and PD. We identified 63 genera only presented in the oral virome (subgingival plaques and saliva), 111 genera only presented in the gut virome, and 87 genera presented in both the oral and gut viromes (Fig. 5A). Subsequently, we analyzed the top 30 genera of these 87 genera coexisting in the oral cavity and the gut. In general, the relative abundances of the top 30 shared genera showed the same trend in oral and gut sites. Namely, genera abundant in oral samples were also abundant in the gut, and genera less enriched in oral samples were less enriched in the gut (Fig. 5B). Importantly, differences were observed among the four groups. *Pepyhexavirus* in subgingival plaques from the HTNnPD group was significantly lower compared with the nHTNnPD control group, and *Pepyhexavirus* in feces was also significantly lower in the HTNnPD group compared with the nHTNnPD group (Fig. 5B). The trends of changes of *Alphabaculovirus*, *Marseillevirus*, and *Emaravirus* in subgingival plaques were similar to those in the gut (Fig. 5B). These results suggested that the oral virome may be transferred to the gut, especially *Pepyhexavirus*, and that specific oral viruses may affect the gut virome composition.

We then evaluated the correlations between the top 30 shared genera in the oral cavity and those in the gut. There were more correlations between gut viruses and salivary viruses than those between gut viruses and subgingival viruses (Fig. 5C and D). In subgingival plaques, *Lymphocryptovirus* had the largest numbers of correlations with gut viruses, including positive correlations with *Chlorovirus*, *Moineauvirus*, *Brussowvirus*, *Roseolovirus*, *Fromanvirus*, and *Avipoxvirus* (Fig. 5C). Salivary *Marseillevirus* was significantly associated with seven gut viruses (Fig. 5D). Moreover, the relative abundance of *Pepyhexavirus* in the gut was significantly correlated with *Avipoxvirus* and *Moineauvirus* of subgingival plaques as well as positively correlated with salivary *Cheoctovirus* (Fig. 5C and D). *Bingvirus* in the gut was positively correlated with *Cheoctovirus* from both subgingival plaques and saliva (Fig. 5C and D). Gut *Pamexvirus* was negatively associated with *Kenoshavirus* and *Pepyhexavirus* from both subgingival plaques and saliva (Fig. 5C and D). These results indicated potential cross-talk between the oral and gut viromes.

### Altered viral-bacterial transkingdom interactions in HTN and PD

**Fig 6:**
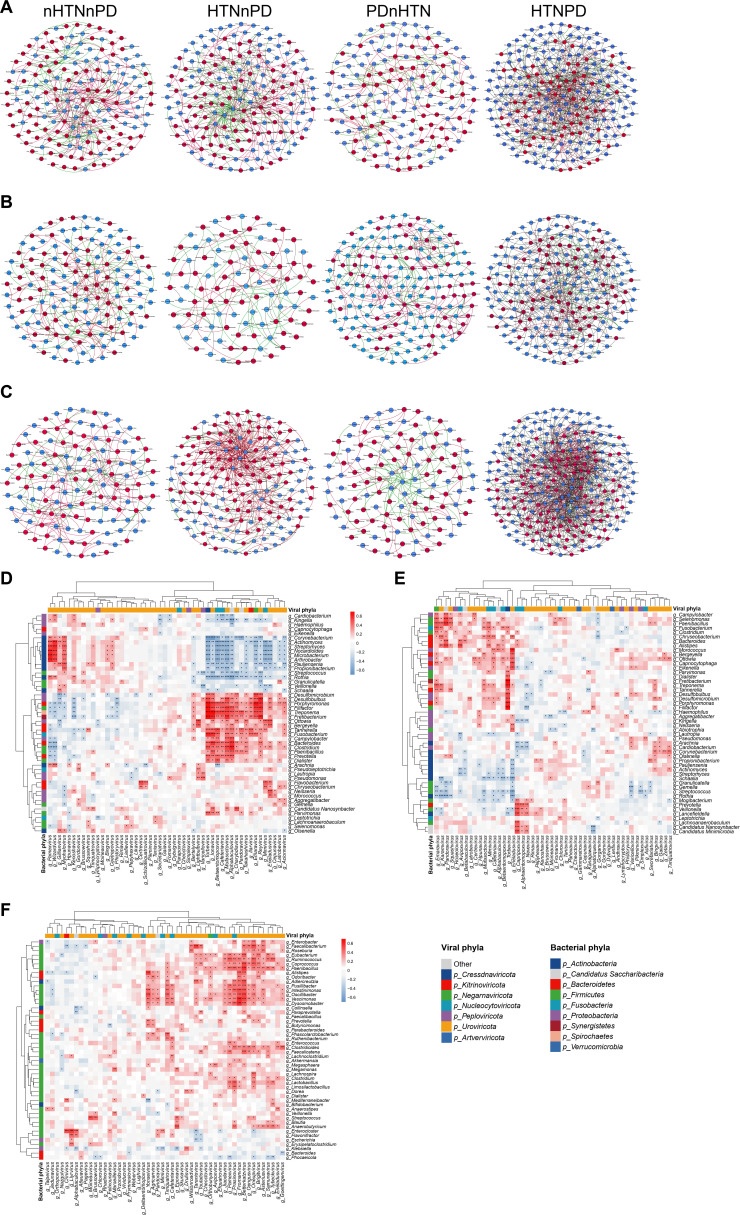
Altered viral-bacterial transkingdom interactions in HTN and PD. (**A–C**) Correlation networks between viruses and bacteria at the genus level in subgingival plaques (**A**), saliva (**B**), and feces (**C**) of participants with or without HTN and/or PD. Red dots indicate bacteria and blue dots indicate viruses. Red lines indicate significant positive correlations, green lines indicate significant negative correlations, and gray lines indicate nonlinear correlations. (**D–F**) Heatmaps of Spearman’s correlation coefficients between relative abundances of the top 50 viral genera in subgingival plaques (**D**), saliva (**E**), and feces (**F**) and those of the top 50 bacterial genera within the same sample type. *n* = 14, 16, 10, and 20 for the nHTNnPD, HTNnPD, PDnHTN, and HTNPD groups, respectively in A–C. *n* = 60 in D–F. Maximal information coefficient (MIC) and Spearman’s correlation coefficients were used for statistical analysis in A–C. Depicted are the viral-bacterial correlation networks with MIC ≥0.45 and *P* value ≤0.05. Spearman’s correlation coefficients between viruses and bacteria >0.5 were identified as positive correlations, less than −0.5 as negative correlations, and the rest as nonlinear correlations. Spearman’s correlation coefficients were used for statistical analysis in D–F. *P < 0.05, **P < 0.01, ***P < 0.001.

In recent decades, many studies have reported the importance of bacteria in the occurrence and development of HTN and PD (17, 28–30), but few studies have focused on viruses. In the present study, we investigated whether viruses and bacteria cooperate and play a larger role in HTN and PD. We evaluated the correlations between bacteria and viruses in the oral cavity and the gut. Overall, virus-bacterium interactions occurred most frequently in subgingival plaques and feces, especially the former (Fig. S4). More communication was observed when we analyzed virus-bacterium interactions using all four groups. Across the three sample sites, the largest number of virus-bacterium interactions was observed in the HTNPD group compared with the other groups (Fig. 6A through C). In subgingival plaques and feces, the nHTNnPD and HTNnPD groups had significantly more virus-bacterium associations than the PDnHTN group (Fig. 6A and C). In saliva, a larger number of virus-bacterium interactions were found in the nHTNnPD and PDnHTN groups than in the HTNnPD group (Fig. 6B). Among these three sites and four groups, most virus-bacterium interactions were significantly positive correlations, except more significantly negative correlations were observed in the saliva of the HTNnPD group and in the feces of PDnHTN and HTNPD groups (Table S2).

We then examined correlations between the 50 most abundant viruses and the 50 most abundant bacteria at the genus level using Spearman correlation analyses (Fig. 6D through F). In subgingival plaques, *Varicellovirus*, a virus positively correlated with SBP, showed a significantly positive correlation with *Lautropia* (Fig. 6D), which was a bacterium significantly increased in HTN subjects (13, 31). *Gillianvirus*, *Liefievirus*, and *Pepyhexavirus*, which were reduced genera in HTN subjects, were significantly correlated with some HTN-related bacteria. *Gillianvirus* was positively correlated with *Olsenella* but negatively correlated with *Desulfomicrobium*, and *Pepyhexavirus* was positively correlated with *Selenomonas* but negatively correlated with *Lautropia* (Fig. 6D). According to a previous study (13), *Olsenella* and *Selenomonas* are significantly decreased in HTN, while *Desulfomicrobium* and *Lautropia* are enriched in HTN. Consistent with subgingival plaques, HTN-enriched salivary *Torbevirus* was negatively associated with salivary *Veillonella* and *Streptococcus* (Fig. 6E), and *Veillonella* and *Streptococcus* are both reduced bacteria in HTN (13, 32, 33). In the present study, most correlations between viruses and bacteria were positive in feces (Fig. 6F). HTN-enriched *Drulisvirus* was positively associated with fecal *Klebsiella* (Fig. 6F). *Oengusvirus*, which was reduced in HTN, was positively correlated with *Faecalibacterium*, *Roseburia*, *Coprococcus*, and *Oscillibacter* (Fig. 6F), all of which bacteria have been reported to be decreased in HTN (34, 35). Additionally, a number of significant correlations between bacteria and *Betterkatzvirus*, which was positively associated with DBP, were observed (Fig. 6F).

From the perspective of PD, *Torbevirus*, *Ionavirus*, *Betaentomopoxvirus*, *Cafeteriavirus*, *Mufasoctovirus*, *Alphabaculovirus*, *Charybdisvirus*, *Pandoravirus*, *Takahashivirus*, *Ilarvirus*, *Emaravirus*, *Acionnavirus*, and *Reyvirus* in subgingival plaques showed strong positive correlations with *Porphyromonas gingivalis*, *Tannerella forsythia*, and *Treponema denticola* (Fig. 6D); these three bacteria were all classical pathogens of PD (36). Similarly, *Torbevirus*, *Cafeteriavirus*, *Emaravirus*, *Karamvirus*, and *Mufasoctovirus* in saliva were positively correlated with red-complex genera (Fig. 6E). Combined with the results of Fig. 2G, *Takahashivirus*, *Cafeteriavirus*, *Ilarvirus*, and *Reyvirus* were significantly increased in subgingival plaques of the PDnHTN group. *Torbevirus* was significantly enriched in the saliva of the PDnHTN group. According to the results of Fig. 4B, *Cafeteriavirus* in subgingival plaques as well as *Torbevirus* and *Andhravirus* in saliva, which were positively associated with IL-6 or CRP, may aggravate PD. In conclusion, these results indicated that these repeatedly appearing viruses, namely, *Takahashivirus*, *Cafeteriavirus*, *Ilarvirus*, and *Reyvirus* in subgingival plaques as well as *Torbevirus* in saliva, may synergize with PD-related bacteria and contribute to the development of PD.

## DISCUSSION

The microbiota plays a profound role in both HTN and PD, and PD exacerbates the development of HTN by oral microbiota and systemic inflammation. Previous studies have mainly focused on depicting the landscape of bacteria in HTN and PD but have rarely focused on the impact of viruses. To the best of our knowledge, the present study is the first to report a comprehensive analysis of the oral and gut viromes in HTN and PD. We collected 180 samples from subgingival plaques, saliva, and feces and performed metagenomic sequencing to assess viral homeostasis among these sites. The present results demonstrated altered biodiversity and community dissimilarity in patients with HTN and/or PD. We also profiled shifts in oral and gut viral compositions and identified associations of the oral and gut viromes with clinical parameters. Moreover, we revealed the communication between the oral and gut viromes and investigated viral-bacterial interactions in HTN and PD. These results may help to understand the effects of the virome on the development of HTN and PD.

The compositions of the oral and gut viromes in patients with HTN and/or PD were different from those in healthy controls. Nine of the top 10 viral families in subgingival plaques and saliva were identical, and five of the top 10 families in the gut were identical to those in the oral cavity. These findings indicated that different sites had different impacts on the composition of dominant viruses and that some closely connected habitats may have more similar compositions. Overall, *Podoviridae*, *Myoviridae*, and *Siphoviridae* were dominant enteroviruses, consistent with previous studies (37, 38). We then compared the virome composition between healthy controls and patients. *Redondoviridae* was significantly increased in patients with PD, which agreed with previous studies (39, 40). The abundance of *Siphoviridae* in subgingival plaques was significantly lower in the PDnHTN group, consistent with the previous study (41). At the genus level, we also analyzed the relative abundances of viruses among the four groups and depicted disease-specific signatures of the oral and gut viromes as follows: for the HTNnPD group, nine genera in the oral cavity and six genera in the gut were significantly changed; for the PDnHTN group, 23 genera in the oral cavity were significantly changed; for the HTNPD group, 14 genera in the oral cavity and three genera in the gut were significantly changed. Thus, the present study reported for the first time the association of these genera with HTN and/or PD. Interestingly, the pathogenicity of some genera in other diseases has been revealed. For example, *Betatorquevirus* is associated with encephalitis/meningoencephalitis and may be linked with certain hematonoses (42, 43). Furthermore, the abundance of *Alphatorquevirus* is associated with posttransplant infection (44, 45).

We next explored the correlations between the oral/gut viromes with BP and other clinical parameters, which elucidated potential pathogenic viruses. In subgingival plaques, *Gillianvirus* was negatively correlated with SBP, and it was significantly decreased in both the HTNnPD group and the HTNPD group, suggesting that *Gillianvirus* in subgingival plaques was negatively correlated with HTN. In saliva, *Torbevirus* was positively correlated with DBP and CRP, and it was significantly increased in both the HTNnPD group and the HTNPD group, indicating that *Torbevirus* in saliva was positively correlated with HTN. Similarly, we concluded that *Likavirus* in subgingival plaques was negatively associated with PD, and *Cafeteriavirus* was positively associated with PD. In addition, salivary *Torbevirus* and *Andhravirus* were positively associated with PD. A member of *Andhravirus* has been reported to prevent *Staphylococcus ureilyticus* infections and the formation of bacterial biofilms (46). Thus, *Andhravirus* may prevent the growth of some oral beneficial bacteria and contribute to the development of PD. Lymphocytes, neutrophils, and eosinophils are all critical for the progression of HTN (47–49). In the present study, we found that lymphocyte, neutrophil, and eosinophil counts were positively associated with the following viruses: *Betaentomopoxvirus* and *Torbevirus* in subgingival plaques; *Torbevirus*, *Tanisvirus*, *Squashvirus*, *Bingvirus*, and *Betaentomopoxvirus* in saliva; *Pagevirus* in feces. These results suggested that these viruses are harmful for HTN.

Because microbial interactions between the oral cavity and gut occur frequently and the interactions may affect HTN and many other diseases (27, 50, 51), we analyzed communications between the oral and gut viromes. Approximately one-third of oral viruses were prevalent in the gut, and *Pepyhexavirus* from subgingival plaques may be transferred to the gut. *Alphabaculovirus*, *Marseillevirus*, and *Emaravirus* in subgingival plaques were also potential oral-gut transmitters. We evaluated the correlations of the top 30 shared genera between the oral cavity and gut but did not find significant correlations between the oral cavity and gut for the same genera. Gut *Pepyhexavirus* was positively correlated with *Avipoxvirus* in subgingival plaques and *Cheoctovirus* in saliva, indicating that oral *Avipoxvirus* and *Cheoctovirus* may promote oral *Pepyhexavirus* transfer to the gut. To date, *Avipoxvirus* has not been reported to cause clinical diseases in humans, and it has been used as a vaccine vector (52, 53).

Close interactions of the virome and bacteriome have been reported in many diseases, including rheumatoid arthritis (54), inflammatory bowel disease (55), and alcoholic hepatitis (56). Because bacteria play an important role in the development of HTN and PD, we further evaluated viral-bacterial interactions in the oral cavity and gut. Viral-bacterial transkingdom interactions were significantly changed in patients with HTN and/or PD compared with healthy controls. The HTNPD group had the most interactions, indicating that HTN and PD synergize to promote communications between the virome and bacteriome. Specifically, we found that many HTN-related viruses were significantly associated with HTN-related bacteria, for example, *Varicellovirus* with *Lautropia*, *Gillianvirus* with *Olsenella*, and *Pepyhexavirus* with *Selenomonas* in subgingival plaques; *Torbevirus* and *Veillonella* with *Streptococcus* in saliva; and *Drulisvirus* with *Klebsiella* in gut. These results further supported the importance of these viruses in the progression of HTN and suggested that viruses and bacteria may cooperate to affect BP. To the best of our knowledge, no studies regarding the associations of these viruses and HTN have been reported. It has been reported that one member of *Varicelloviruses*, namely, varicella-zoster virus, causes varicella, and its reactivation leads to neurological disease in elderly and immunocompromised individuals (57, 58). Similarly in PD, we also found that many viruses were significantly correlated with classical red-complex bacteria, which are known pathogens that cause PD (59, 60). *Takahashivirus*, *Cafeteriavirus*, *Ilarvirus*, and *Reyvirus* in subgingival plaques as well as *Torbevirus* in saliva may synergize with red-complex bacteria and other pathogens to aggravate PD. *Pandoravirus* has been isolated from inflamed eyes of patients with keratitis (61, 62), showing its contribution to inflammation.

The present study first revealed significant alterations in the oral and gut viromes in HTN and/or PD. We also identified key indicator viruses and their clinical implications in HTN or PD. Viruses and bacteria may cooperate to affect HTN and PD. Correspondingly, HTN and PD may synergize to improve communications between viruses and bacteria. The present findings improved the overall understanding of the viromes in HTN and PD, providing vital insights into the potential role of the virome in the development of HTN and PD. To further elucidate the role of the oral and gut viromes in the development process, more cohort studies and animal experiments are needed.

## MATERIALS AND METHODS

### Enrollment and sample collection

Subjects in this study were recruited from the Department of Cardiology at Shanghai Ninth People’s Hospital. They were individually told about the information of the study, including its risks and benefits. Subjects who decided to participate this study signed the informed consent. Then, their BP were measured, and their oral conditions were checked. The diagnosis of HTN was in accordance with the 2018 ESC/ESH Guidelines with SBP ≥140 mmHg and/or DBP ≥90 mmHg (63). The diagnosis of PD was based on the standards of the Centers for Disease Control and Prevention/American Academy of Periodontology case definitions, namely, at least two interproximal sites with clinical attachment loss ≥3 mm and probing depth ≥4 mm (64). The exclusion criteria were as follows: pregnancy; smoking; diseases, including peripheral artery disease, autoimmune disease, heart failure, renal failure, cancer, irritable bowel syndrome, inflammatory bowel disease, and recurrent aphthous oral ulcers; taking antibiotics or probiotics within the last 2 months; subjected to oral or gut surgeries or treatments within the last 2 months; and having less than eight natural teeth. Feces samples were collected first, and then, saliva and subgingival plaques were collected within two hours. Feces were freshly collected with stool collection containers. Participants were informed to avoid eating, drinking, or brushing their teeth before collecting oral samples. The unstimulated saliva of each participant was collected by drooling into a 50-mL sterile centrifuge tube within 15 min, and the volume of saliva was more than 5 mL. The saliva was then rapidly preserved in Saliva DNA Preservation Solution (Huayueyang Biotech, Beijing, China). Subgingival plaques were collected using Hu-Friedy subgingival curettes and preserved in phosphate-buffered saline containing 20% glycerol. Subgingival plaques, saliva, and feces were transported to the laboratory on ice within 2 hours after collection and stored at −80°C for DNA extraction.

### DNA extraction and metagenomic sequencing

Total genomic DNA was extracted using the OMEGA Soil DNA Kit (M5635-02, Omega Bio-Tek, Norcross, GA, USA) according to the manufacturer’s instructions. The concentration and purity of extracted DNA were monitored by a NanoDrop NC2000 spectrophotometer (Thermo Fisher Scientific, Waltham, MA, USA) and 1% agarose gel electrophoresis, respectively.

Metagenomic sequencing was performed using whole genome shotgun sequencing on an Illumina NovaSeq platform at Personal Biotechnology Co., Ltd. (Shanghai, China) with 150-bp paired-end reads and a 400-bp insert size. All raw reads were quality controlled by discarding low-quality reads and removing adapters by fastp (v0.23.2) (65). Reads were further aligned to the human genome using Bowtie2 to remove host-derived contamination. Clean reads were then used for *de novo* assembly to construct the metagenome. Taxonomic annotation was performed using Kraken2 (66). Bracken was further used to perform statistical analysis on the results from Kraken2.

### Statistical analysis

Alpha and beta diversities were calculated by the diversity function and PCoA function with the vegan package (2.6-2) and ape package (5.6-2), respectively, in R. The Vegdist function in the vegan (2.6-2) package in R was used to determine the Bray‒Curtis distance. MaAsLin2 was used to test differentially abundant viruses adjusted for age, gender, and body mass index. Differentially abundant viruses were finally identified by the overlap outputs of limma voom, MaAsLin2, and Wilcoxon test. Data processing and visualization were performed by the ggplot2 (3.3.6), pheatmap (1.0.12), and UpSet (1.4.0) packages in R. The Wilcoxon rank-sum test was used for statistical analysis between groups. Multiple-comparison adjustment was implemented by a false discovery rate procedure with the Benjamini-Hochberg method. The core viromes in each group were identified as an occurrence over 80% in that group. Correlations between the virome and clinical parameters as well as correlations between the oral and gut viromes were performed using the Spearman correlation coefficient. Virome and bacteria correlations were tested by the MIC scores and Spearman correlation (67). The network diagram was performed by the Gephi software (version 0.9.2). All statistical tests were performed using R 4.1.1 software, and a *P* value of <0.05 was considered statistically significant.

## Data Availability

Raw sequences are available in the Sequence Read Archive of NIH (BioProject No. PRJNA765566). The clinical metadata are available in Data File S1.

## SUPPLEMENTAL MATERIAL

Data File S1 msystems.01169-23-s0001.xlsx Clinical metadata.

Supplemental material msystems.01169-23-s0002.docx Fig. S1 to S3 and Tables S1 to S2.

ASM does not own the copyrights to Supplemental Material that may be linked to, or accessed through, an article. The authors have granted ASM a non-exclusive, world-wide license to publish the Supplemental Material files. Please contact the corresponding author directly for reuse.
