# Supplementary material for: Alterations of oral and gut viromes in hypertension and/or periodontitis
Source: mSystems. 2023 Dec 18;9(1):e01169-23. doi: 10.1128/msystems.01169-23 (PMC10804974; doi:10.1128/msystems.01169-23)
Supplement: Supplemental material — Fig. S1 to S3 and Tables S1 to S2. [file msystems.01169-23-s0002.docx]

**Figure S1**


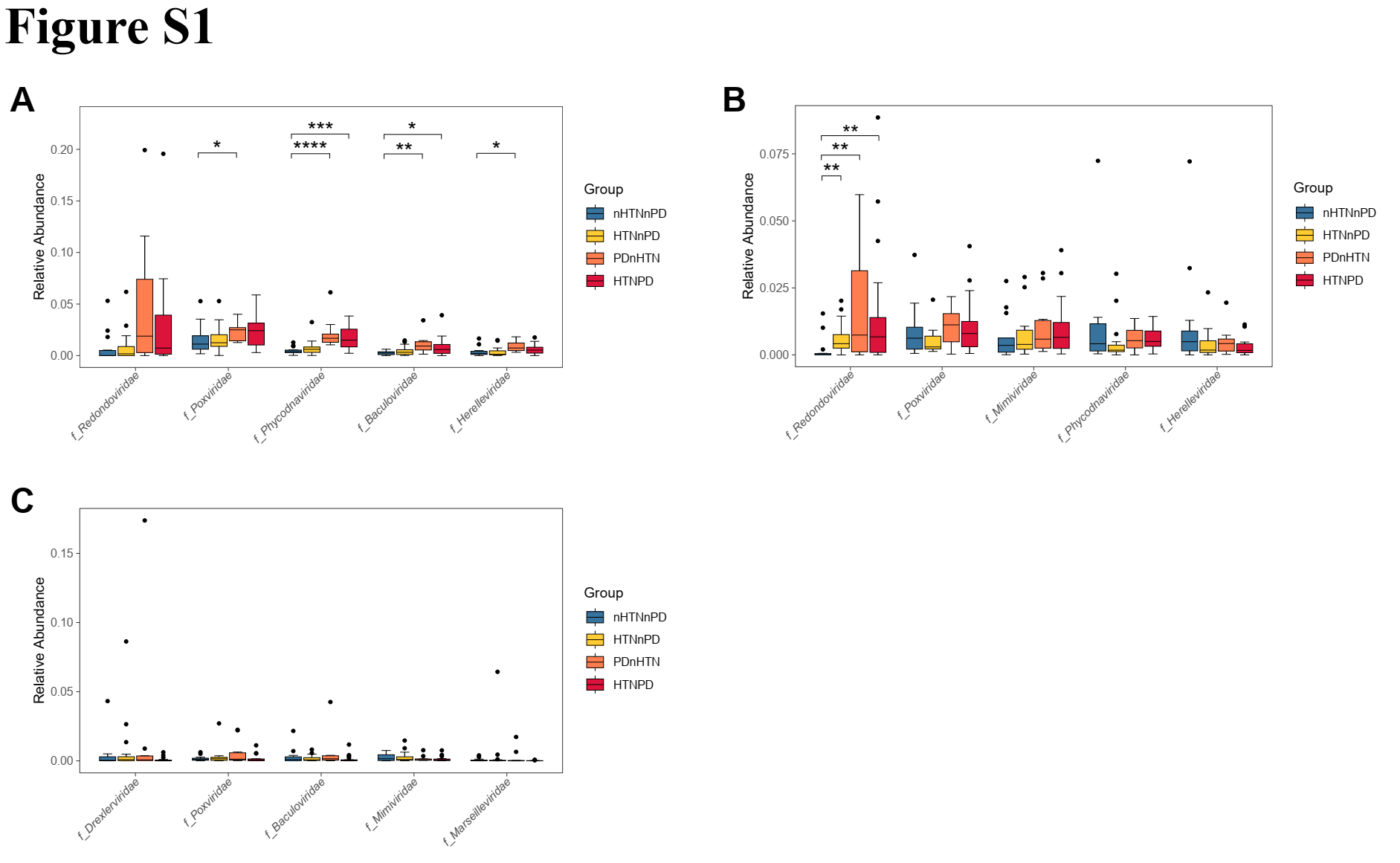


**Fig. S1.** **Alterations of oral and gut viral composition at the family level in patients with HTN and/or PD.** (A-C) Analyses of relative abundances of the top 6-10 viral families in subgingival plaques (A), saliva (B) and feces (C). n = 14:16:10:20 for the nHTNnPD, HTNnPD, PDnHTN and HTNPD group, respectively. Wilcoxon rank-sum test was used for statistical analysis. *p < 0.05, **p < 0.01, ***p < 0.001.

**Figure S2**


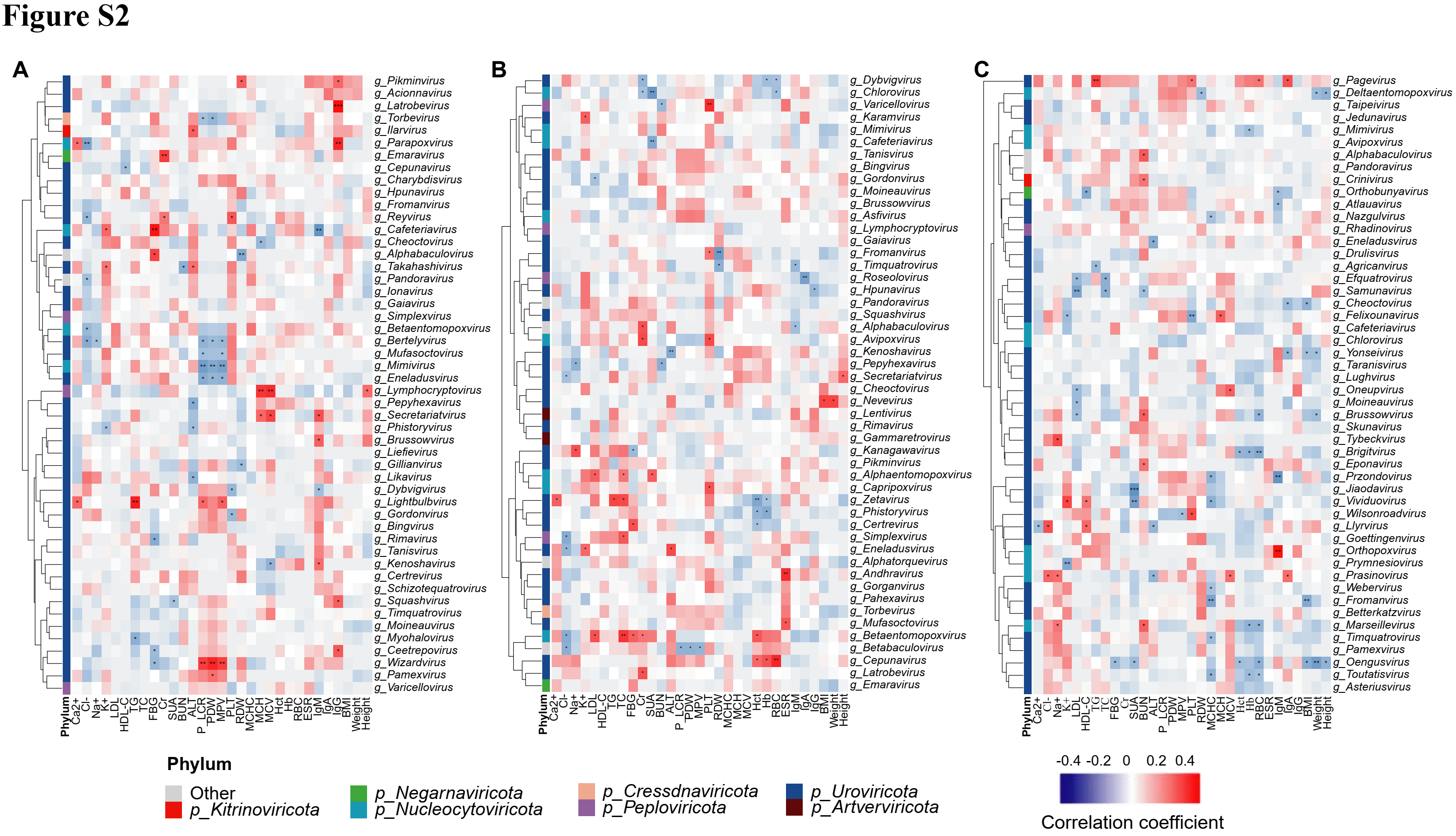


**Figure S2. Associations between oral/gut virome and other clinical parameters.** (A-C) Heatmaps of Spearman’s correlation coefficients between other clinical parameters (electrolytes, metabolic traits, liver and kidney function, platelets, red blood cells, immunoglobulins and anthropometric measurements) and relative abundances of the top 50 viral genera of subgingival plaques (A), saliva (B) and feces (C). Ca2+: calcium, Cl-: chlorine, Na+: sodium, K+: potassium, LDL: low-density lipoprotein cholesterol, HDL-C: high-density lipoprotein cholesterol, TG: triglyceride, TC: total cholesterol, FBG: fasting blood glucose, Cr: creatinine, SUA: serum uric acid, BUN: blood urea nitrogen, ALT: alanine aminotransferase, P-LCR: platelet-large cell ratio, PDW: platelet distribution width, MPV: mean platelet volume, PLT: platelet, RDW: red blood cell distribution width, MCHC: mean corpuscular hemoglobin concentration, MCH: mean corpuscular hemoglobin, MCV: mean corpuscular volume, Hct: hematocrit, Hb: hemoglobin, RBC: red blood cells, ESR: erythrocyte sedimentation rate, Ig: Immunoglobulin, BMI: body mass index. n = 60. *p < 0.05, **p < 0.01, ***p < 0.001.

**Figure S3**


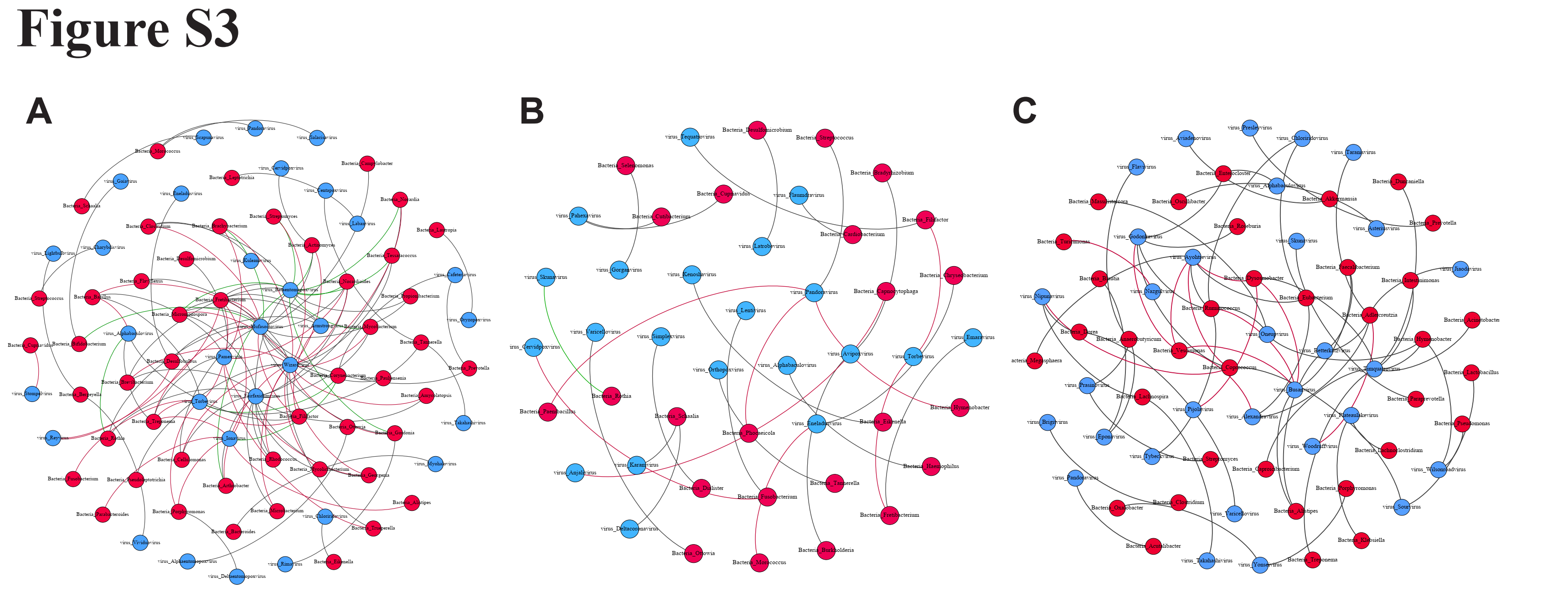


**Figure S3.** **Viral-bacterial trans-kingdom interactions in the oral cavity and the gut of participants with or without HTN and/or PD.** (A-C) Overview of correlation networks between viruses and bacteria at the genus level within subgingival plaques (A), saliva (B), and feces (C). Red dots indicate bacteria and blue dots indicate viruses. Red lines indicate significantly positive correlations, green lines indicate significantly negative correlations, and gray lines indicate nonlinear correlations. n = 60. Maximal information coefficient (MIC) and Spearman’s correlation coefficients were used for statistical analysis. Depicted are the viral-bacterial correlation networks with MIC ≥ 0.45 and P value ≤ 0.05. Spearman’s correlation coefficients between viruses and bacteria > 0.5 were identified as positive correlations, < -0.5 as negative correlations, and the rest as nonlinear correlations.

**Table S1. The detailed demographics and clinical parameters of participants.**

|  | nHTNnPD | HTNnPD | PDnHTN | HTNPD | P value |
| --- | --- | --- | --- | --- | --- |
| Sex, n (%) |  |  |  |  |  |
| Male | 4 (28.57%) | 8 (50%) | 4 (40%) | 9 (45%) | 0.67 |
| Female | 10 (71.43%) | 8 (50%) | 6 (60%) | 11 (55%) |  |
| Age (years) | 66.79 ± 9.75 | 68.81 ± 5.96 | 68.70 ± 6.33 | 67.05 ± 5.40 | 0.79 |
| Height (cm) | 162.43 ± 8.73 | 165.44 ± 7.56 | 162.80 ± 6.68 | 163.55 ± 6.48 | 0.69 |
| Weight (kg) | 61.21 ± 7.31 | 65.09 ± 11.13 | 62.92 ± 7.93 | 65.77 ± 8.80 | 0.49 |
| BMI (kg/m^2^) | 23.28 ± 2.19 | 23.69 ± 3.21 | 23.74 ± 2.63 | 25.05 ± 3.81 | 0.37 |
| SBP (mmHg) | 120.48 ± 13.54 | 125.12 ± 16.87 | 131.57 ± 15.64 | 131.12 ± 16.39 | 0.19 |
| DBP (mmHg) | 74.43 ± 6.33 | 75.12 ± 7.59 | 78.57 ± 8.55 | 79.62 ± 8.05 | 0.17 |
| Probing depth (mm) | <4 | <4 | ≥4 | ≥4 | NA |
| Attachment loss (mm) | <3 | <3 | ≥3 | ≥3 | NA |

Definition of abbreviations: BMI, body mass index; NA, not available. Data are shown as mean ± SD or n (%). Pearson’s chi-square test was used for statistical analyses of sex. One-way analysis of variance (ANOVA) was used for statistical analyses of age, height, weight, BMI, and blood pressure.

**Table S2. The number of positive and negative correlations between viruses and bacteria.**

**a. The number of positive and negative correlations in subgingival plaques.**

|  | nHTNnPD | HTNnPD | PDnHTN | HTNPD |
| --- | --- | --- | --- | --- |
| nonlinear correlations | 37 | 63 | 0 | 373 |
| positive correlations | 140 | 143 | 92 | 118 |
| negative correlations | 99 | 112 | 88 | 62 |
| Total | 276 | 318 | 180 | 553 |

**b. The number of positive and negative correlations in saliva.**

|  | nHTNnPD | HTNnPD | PDnHTN | HTNPD |
| --- | --- | --- | --- | --- |
| nonlinear correlations | 27 | 23 | 4 | 288 |
| positive correlations | 91 | 35 | 122 | 70 |
| negative correlations | 74 | 50 | 109 | 41 |
| Total | 192 | 108 | 235 | 399 |

**c. The number of positive and negative correlations in feces.**

|  | nHTNnPD | HTNnPD | PDnHTN | HTNPD |
| --- | --- | --- | --- | --- |
| nonlinear correlations | 33 | 75 | 1 | 685 |
| positive correlations | 83 | 262 | 52 | 40 |
| negative correlations | 43 | 22 | 73 | 203 |
| Total | 159 | 359 | 126 | 928 |
